# Phosphorylcholine Phosphatase: A Peculiar Enzyme of *Pseudomonas aeruginosa*

**DOI:** 10.4061/2011/561841

**Published:** 2011-09-11

**Authors:** Carlos Eduardo Domenech, Lisandro Horacio Otero, Paola Rita Beassoni, Angela Teresita Lisa

**Affiliations:** Departamento de Biología Molecular, Área Bioquímica, Facultad de Ciencias Exactas, Fisicoquímicas y Naturales, Universidad Nacional de Río Cuarto, Ruta Nacional 36 Km 601, 5800 Río Cuarto, Córdoba, Argentina

## Abstract

*Pseudomonas aeruginosa* synthesizes phosphorylcholine phosphatase (PchP) when grown on choline, betaine, dimethylglycine or carnitine. In the presence of Mg^2+^ or Zn^2+^, PchP catalyzes the hydrolysis of *p*-nitrophenylphosphate (*p*-NPP) or phosphorylcholine (Pcho). The regulation of *pchP* gene expression is under the control of GbdR and NtrC; dimethylglycine is likely the metabolite directly involved in the induction of PchP. Therefore, the regulation of choline metabolism and consequently PchP synthesis may reflect an adaptive response of *P. aeruginosa* to environmental conditions. Bioinformatic and biochemistry studies shown that PchP contains two sites for alkylammonium compounds (AACs): one in the catalytic site near the metal ion-phosphoester pocket, and another in an inhibitory site responsible for the binding of the alkylammonium moiety. Both sites could be close to each other and interact through the residues ^42^E, ^43^E and ^82^YYY^84^. Zn^2+^ is better activator than Mg^2+^ at pH 5.0 and it is more effective at alleviating the inhibition produced by the entry of Pcho or different AACs in the inhibitory site. We postulate that Zn^2+^ induces at pH 5.0 a conformational change in the active center that is communicated to the inhibitory site, producing a compact or closed structure. However, at pH 7.4, this effect is not observed because to the hydrolysis of the [Zn^2+^L_2_^−1^L_2_^0^(H_2_O)_2_] complex, which causes a change from octahedral to tetrahedral in the metal coordination geometry. This enzyme is also present in *P. fluorescens*, *P. putida*, *P. syringae*, and other organisms. We have recently crystallized PchP and solved its structure.

## 1. Introduction

Bacteria possess multiple proteins with the capacity to catalyze the hydrolysis of phosphoric esters in either acidic or alkaline media. In addition to having different optimal pH values for their activity, these enzymes differ in their dependence on metal ions, which are generally divalent cations found in the second or third period of the periodic table. In our previous review, we summarized work showing that the synthesis of *Pseudomonas aeruginosa* phosphorylcholine phosphatase (PchP) in the presence of low or high orthophosphate concentration depends on choline, betaine, dimethylglycine, or carnitine added to the culture medium as the carbon and/or nitrogen source. In addition, the gene for PchP was located, and the most current information on the kinetic, biochemical, biophysical, and molecular characteristics of PchP was summarized [1]. Phosphorylcholine (Pcho), phosphorylethanolamine, and *p*-nitrophenylphosphate (*p*-NPP), substrates of PchP, are hydrolyzed to orthophosphate plus choline, ethanolamine, and *p*-nitrophenol, respectively [2, 3]. Because *P*. *aeruginosa *is an opportunistic Gram-negative bacterium capable of producing infections at different levels in higher organisms, we dedicated our effort toward in-depth characterization of PchP. Therefore, this paper focuses on fundamental studies at the molecular, biochemical, bioinformatic, and biophysical levels, with the goal of compiling all current information on PchP.

## 2. Molecular Studies

PA5292 was identified as the locus encoding PchP in the *P. aeruginosa* PAO1 genome using various microbiological and molecular experiments [4]. After this identification our interest was focused on the regulation of *pchP* gene expression. Wargo et al. reported that the induction of *pchP* transcription by glycine betaine, a product of choline oxidation, via betaine aldehyde, is mediated by GbdR, an AraC family transcription factor [5]. The same authors also described GbdR as a specific regulator of genes involved in choline metabolism [6]. The construction of a Δ*gbd*R strain allowed us to confirm the direct impact of GbdR on choline catabolic genes because no growth was detected with this mutant in media with choline, choline/NH_4_^+^, or choline/succinate [7]. In addition, with wild-type *P. aeruginosa*, we have also observed that choline, betaine, dimethylglycine, or carnitine (which is metabolized to betaine) utilization was absolutely necessary for the synthesis of PchP [8–10]. In addition, it is known that in the absence of preferred sources, a two-component system comprising the global regulators NtrBC and CbrAB is activated by the depletion of nitrogen and carbon in many bacteria including *P. aeruginosa *[11–13]. As choline and the various amino acids are not typically preferred carbon or nitrogen sources, we hypothesized that besides GbdR, enzymes whose synthesis is dependent on choline catabolism might be also under the control of NtrBC and/or CbrAB. In control experiments with histidine where NtrBC and CbrAB were activated [11, 12], no *β*-galactosidase activity (a measure of *pchP* expression) was detected [7]. The specific induction produced by choline led us to focus other experiments in this direction, utilizing Δ*cbr*B, Δ*ntr*C, and Δ*ntr*C/Δ*cbr*B mutants of *P. aeruginosa* PAO1, containing the construction P1::*lacZ *(7) inserted into the bacterial chromosome. These experiments showed that CbrB is crucial when choline is the only nutrient for growth because it was observed that the Δ*cbr*B strain did not grow in media containing choline as a carbon or carbon/nitrogen source [7]. With choline as nitrogen source and succinate as carbon source, growth of the Δ*cbr*B mutant was evident, and the increase of *β*-galactosidase activity was similar to the observed in the wild-type strain [7]. Contrary to experiments with the Δ*cbr*B mutant, the Δ*ntr*C strain did not grow in a choline/succinate medium but did grow in media containing choline as the carbon or carbon/nitrogen source. In these two last culture media experiments, the *β*-galactosidase activity was reduced by only 65% with respect to the wild-type strain [7]. The 35% of remnant activity suggested that other regulators besides NtrC might be participating in the synthesis of PchP, but this point has not yet been resolved. 

However, experiments with choline or dimethylglycine as carbon and nitrogen sources by *P. aeruginosa *PAO1 P1::*lacZ* showed the rapid response of the promoter during the beginning of the lag phase of growth; the production of *β*-galactosidase activity, at the maximum point, was approximately 50% higher in dimethylglycine medium than in choline medium [16]. These results together with findings from other laboratories [5, 6] demonstrated that GbdR and CbrB are indispensable for choline metabolism when it was the carbon source. In addition, GbdR and NtrC are necessary for the expression of PchP; dimethylglycine is likely the metabolite directly involved in the induction of PchP. Therefore, the regulation of choline metabolism and consequently PchP synthesis may reflect an adaptive response of *P. aeruginosa *to environmental conditions. This is in line with some results described in* P. fluorescens*, where a similar pattern of regulation for the catabolism of certain amino acids (histidine, proline, leucine, isoleucine, and valine) is also controlled by CbrAB and NtrBC. The specific induction of enzymes involved in their metabolism is indispensable in the presence of the specific substrate [13]. 

In order to explain the participation of specific regulator GbdR and the global regulators NtrBC and CbrAB in the synthesis of PchP, we first focused our attention on the transcriptional organization of the *pchP* gene [7]. Bioinformatic predictions, confirmed experimentally by site-directed mutagenesis and transcriptional fusion analyses, led to the conclusion that full *pchP *expression depends on an upstream region located −188 to −68 bp from the ATG start codon. Although this sequence has a score below the best prediction for a *σ* ^54^-dependent promoter, it contains (i) the conserved −24 GG and −12 GC elements characteristic of putative *σ* ^54^-dependent promoters, (ii) a region that resembles an IHF binding site, and (iii) a potential EBP binding site resembling the palindromic NtrC-binding consensus site [17, 18]. The dependence of *pchP* expression on the *σ* ^54^ factor was confirmed using a Δ*rpo*N mutant strain, which showed a strong reduction in expression (*≅*70%). On this point, we assumed that the residual expression may result from a second promoter that could be activated by GbdR. Because the upstream region of PA5380 (*gbdR*), as analyzed by PromScan software, has a high similarity score (86 out of 100) with *σ* ^54^-dependent promoters, we suggest that a cascade of events happen when choline is the alternative source of carbon, nitrogen, or carbon and nitrogen; (i) NtrB and CbrA respond to the absence or deficiency of the preferred carbon or nitrogen sources. (ii) These sensors activate, through phosphorylation, the respective response regulators NtrC and CbrB, which are enhancers of *σ* ^54^-dependent promoters. (iii) As choline is an alternative carbon and nitrogen sources for growth, all genes involved in its catabolism or related to choline catabolism, for example, *gbdR, pchP* (and some other) are first activated. (iv) Once the intracellular concentration of GbdR increased, the enzymes of choline catabolism are directly activated [6] and specifically interact with the *pchP *promoter near the −12 box [5]. This interaction may move the *σ* ^54^- polymerase from the promoter, so transcription from other promoter sequences can occur. In conclusion, we are only beginning to understand this activity because *pchP *expression now appears much more complex than previously anticipated, suggesting that more than one form of RNA polymerase and multiple transcriptional regulators could be involved.

## 3. Bioinformatic Studies

Initial bioinformatics studies indicated that PchP belongs to the haloacid dehalogenase hydrolase (HAD) superfamily. According to the following reference [[24], (http://www.ncbi.nlm.nih.gov/Structure/cdd/cddsrv.cgi)], “*The haloacid dehalogenase-like superfamily includes L-2-haloacid dehalogenase, epoxide hydrolase, phosphoserine phosphatase, phosphomannomutase, phosphoglycolate phosphatase, P-type ATPase, and many others, all of which use a nucleophilic aspartate in their phosphoryl transfer reaction. All members possess a highly conserved alpha/beta core domain, and many also possess a small cap domain, the fold and function of which is variable. Members of this superfamily are sometimes referred to as belonging to the DDDD superfamily of phosphohydrolases.*” The *pchP* gene published in the *Pseudomonas* genome database _V2_ indicates that it codes for a protein containing 349 amino acids. However, because PchP is exported to the periplasmic space, it produces a mature protein containing 327 amino acids. Therefore, motifs I, II, and III, which are characteristic of enzymes belonging to the HAD superfamily, are found at ^31^ **D**M**D**NT^35^, ^166^S, and ^242^K/^261^G**D**TPDS**D** ^267^ (the aspartyl residues involved in the catalysis of PchP are denoted in bold and underlined) [25, 26]. 

### 3.1. Molecular Modeling

**Figure 1:**
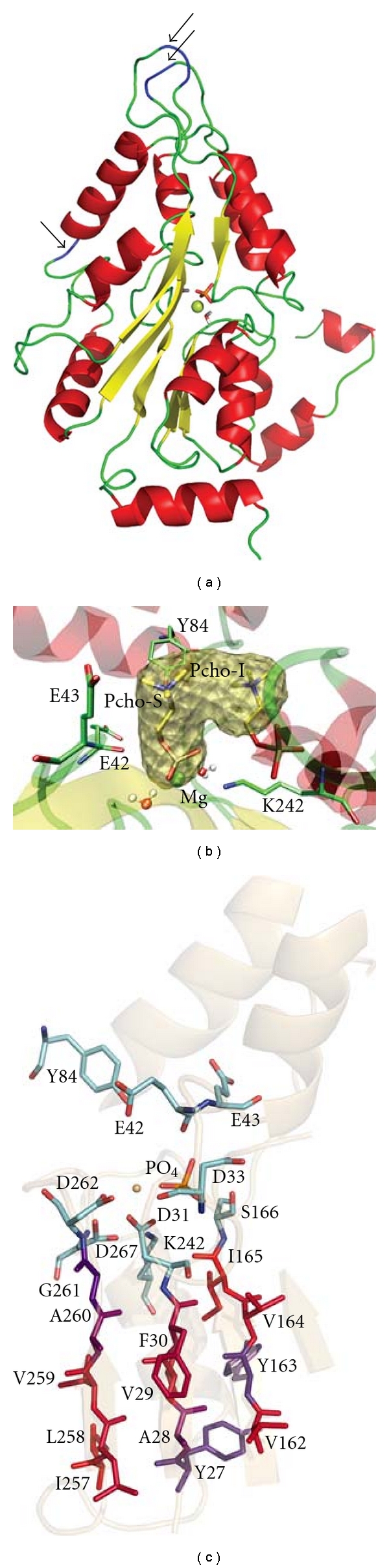
Model of PchP. (a) Cartoon representation. The points where loops were eliminated for modeling are indicated by an arrow. (b) A model of PchP representing the pocket detected by ICM-Pro and two conformations of Pcho. Cartoon representation of the secondary structure, stick representation of certain residues and Pcho, and msms (solvent excluded surface) representation of the pocket. Pcho-S: substrate conformation, Pcho-I: inhibitor conformation. (c) Stick representation of residues from the active site of members of the HAD superfamily, plus the hydrophobic pocket surrounding the active site. The residues of the active site are colored by element, and the rest are colored by hydrophobicity according to the Kyte and Doolittle scale [14], in which the most hydrophobic residues are red, and the most hydrophilic are blue.

As previously indicated [24], all members of the HAD superfamily share a similar catalytic mechanism that uses a nucleophilic aspartate, but the overall homology among these enzymes is small. Their sequence identity is less than 15% and is focused on three short motifs that form the active site [27]. In previous modeling studies of PchP, threading techniques were employed using the *Methanococcus jannaschii *phosphoserine phosphatase atomic coordinates as a template (*mj*PSP, PDB code 1F5S) [28]. This model was very useful for determining the catalytic relevance of the residues in the active site and for the description of phosphate and Mg^2+^ binding [25, 26]. However, the template was much shorter than PchP, and 3d-pssm did not model several regions; consequently the final PchP model was segmented. These problems required major improvements to the model to allow the use of a docking approach to make advances in determining the quaternary ammonium-binding site. Therefore, a strategy was implemented combining protein fold recognition and comparative modeling by satisfaction of spatial restraints. First, the PchP sequence (lacking the signal peptide) was submitted to the PHYRE server [29], which is a more accurate and updated version of 3d-pssm. This server performs alignments with proteins of known structure according to sequence identity, secondary structure as predicted by PSI-PRED, and solvent accessibility. The best-ranked template was phosphoserine phosphatase from *M. jannaschii,* with 100% confidence, but once again, template size was a problem. While *mj*PSP has 222 residues, PchP has 327; this results in regions without a template for PchP modeling. In addition, the secondary structure predicted for those regions was coiled and formed large loops. Therefore, it was not possible to model those loops with an acceptable level of accuracy, and those regions were discarded for the next step (residues 56–67, 120–136, and 206–224). This edited alignment was the input alignment for MODELLER. The final model was minimized and equilibrated using molecular dynamics, and the regions containing the three mega-loops that were eliminated (indicated by arrows) are shown in Figure 1(a). Like all members of the HAD superfamily, the model of PchP possesses an *α*/*β* core domain that consists of a central parallel *β*-sheet flanked by *α*-helices on both sides. The catalytic scaffold is located in this central core, which is composed of four loops. Loop 1 contains the nucleophilic aspartate, loops 2 and 3 contain the serine from motif II and the lysine from motif III, respectively, and loop 4 contains the two aspartate residues of motif III involved in metal coordination. Despite the absence of the three mega-loops, the new model was significantly improved compared to the previous one [25] and allowed docking studies to be performed in the active site region.

### 3.2. Docking Assays

Docking studies with Pcho performed with wild-type PchP (wtPchP) only showed one major conformation for the substrate regardless of whether the entire protein or only the HAD pocket was mapped (Figure 1(b)). The alternative conformations were very similar, with only slightly modified rotation angles. The presence of two interaction sites for Pcho in PchP was measured in crude extracts [30] and in the purified enzyme [3], and attention was focused on finding an explanation for this observation. A second conformation of Pcho was detected when the docking study was performed with the wtPchP-Pcho complex obtained after the first docking. A second molecule of Pcho was docked into the pocket at its alkylammonium moiety in an empty zone of the pocket (Figure 1(b)). The binding energy of the second conformation was found to be much higher (−75.5 Kcal mol^−1^ versus −4.9 Kcal mol^−1^), indicating that the substrate first binds in the catalytic position with high affinity, and only when this site is occupied is a second molecule of substrate able to bind into the pocket in an inhibitory conformation. The inhibitory conformation of the substrate may block the entry and/or exit of the substrate and products; the enzyme-substrate-substrate complex can be productive, but with much lower efficiency. This result explains the inhibition caused by high concentrations of substrate. Considering the pocket shape detected by ICM-Pro, as well as the kinetic and docking results, we hypothesized that the binding pocket is composed of a catalytic site (with a subsite for the phosphate moiety and a subsite for the alkylammonium moiety) together with an inhibitory site capable of recognizing the alkylammonium moiety of Pcho (or another NR_4_^+^ group) (Figure 1(b)). Therefore, it appeared likely that different alkylammonium compounds (AACs) could bind to both sites. Kinetic experiments seemed to confirm this possibility (the data are discussed at the end of the next section).

## 4. Biochemical Studies

**Figure 2:**
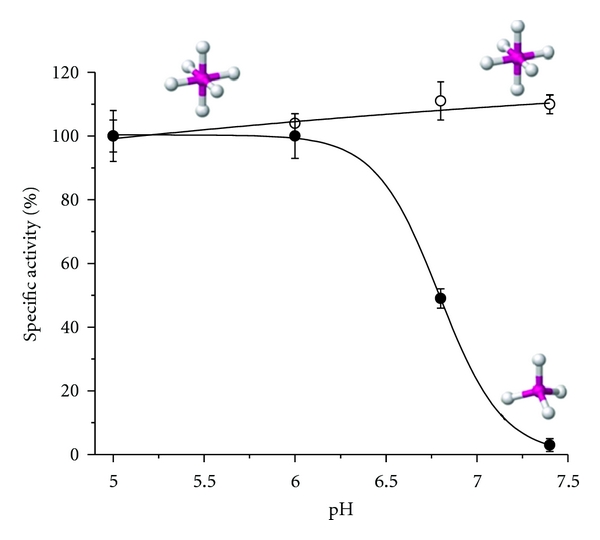
Effect of pH on PchP activity in the presence of 10 mM Mg^2+^ (open circle) or 0.06 mM Zn^2+^ (filled circle). The buffers used were 100 mM HAc/NaAc, pH 5.0, 100 mM HAc/KAc, pH 6.0, 50 mM Hepes/NaOH, pH 6.8, and 50 mM Hepes/NaOH, pH 7.4. Insert shows the coordination sphere for each metal.

PchP was discovered as an acid phosphatase (AcPase) whose activity, measured with *p*-nitrophenylphosphate (*p*-NPP) in the presence of Mg^2+^ at pH 5.0, is inhibited by choline, betaine, acetylcholine, Pcho, and many AACs [8, 9, 15]. These unusual acid phosphatase characteristics, measured in the laboratory [8, 31] or described by other authors [32–36], led to the proposal that the AcPase produced by *P. aeruginosa* grown in the presence of choline or derivative metabolites is capable of catalyzing the hydrolysis of *p*-NPP, Pcho, and phosphoethanolamine [3]. Remarkably, the optimum pH (measured in the presence of Mg^2+^) depends on the substrate used; it is approximately 5.0 and 6.0 for *p*-NPP and phosphoethanolamine, respectively. The optimal pH range for Pcho was found to have a plateau between 5.0 and 8.0 [3]. Considering these data and the fact that PchP acts in the periplasmic space (a compartment where the pH may vary according to environmental conditions) [37], kinetic properties, including saturation by substrate, activation by divalent cations, and inhibition by AACs, were studied with Pcho as the substrate at pH 5.0 and 7.4, and with *p*-NPP as the substrate at pH 5.0 (with this latter substrate, there is no enzymatic activity at pH 7.4). The first studies were performed with a recombinant enzyme, initially as an intein fusion [4, 25, 26] and later with the enzyme expressed with a histidine tag [38]. The catalytic importance of the aminoacyl residues on motifs, I, II, and III was shown through site-directed mutagenesis of the aspartyl (^31^D, ^33^D) and threonyl (^35^T) residues of motif I, of the seryl (^166^S) residue of motif II, and of the lysyl (^242^K), glycyl (^261^G), and aspartyl residues (^262^D, ^265^D, and ^267^D) of motif III (Figure 1(c)) [25]. By comparison with the phosphoserine phosphatase of *M. jannaschii* [28], ^31^D is phosphorylated during phosphoester hydrolysis, and the oxygen atom of the carboxyl group of ^31^D may be involved in nucleophilic attack on the phosphorus atom of the substrate (either *p*-NPP or Pcho). The ^33^D residue is important for catalysis because it participates in the phosphorylation of ^31^D. Considering the motif III residues ^262^D, ^265^D, and ^267^D, it has been shown that ^262^D and ^267^D are the aspartyl residues involved in catalysis. Additionally, it has been shown that the positively charged group of lysyl residue ^242^K is necessary to stabilize the negative charge of the phosphate [25]. After confirming the residues that interact with phosphate and magnesium, we focused our interest on the interaction with the choline moiety of the substrate. This approach was based on comparison to the human enzyme PHOSPHO1 (a phosphocholine/phosphoetanolamine phosphatase also belonging to the HAD superfamily) [39], PHOSPHO2 [40], and choline binding proteins of Gram (+) bacteria [41]. We selected residues ^42^E, ^43^E, and the aromatic triplet ^82^YYY^84^ as possible candidates to interact with Pcho and *p*-NPP. Using a comprehensive approach including homology modeling, site-directed mutagenesis, and kinetic and docking studies, we show that the residues mentioned above interact differently with Pcho and *p*-NPP. We found that ^43^E of PchP is critical for the distinctive inhibition produced by high concentrations of Pcho and that the absence of the side chain in that position makes PchP a more efficient acid phosphatase. ^42^E has relevance in substrate recognition because its substitution with alanine decreased activity with *p*-NPP but increased activity with Pcho. Because mutations in residues ^43^E and the three tyrosines affect both the affinity for substrate and the inhibitory effect for high levels of Pcho, we believe that both a catalytic and regulatory site are present in PchP and that they are near each other in space and even share residues [42]. After identifying the catalytic pocket of PchP through kinetic experiments and developing a single structural model [25, 26], the interactions of the phosphate moiety with the activating metal ions Mg^2+^, Zn^2+^, and Cu^2+^ were investigated [26]. The first results obtained with the recombinant enzyme confirmed previous findings [3, 43]. The new results indicated that at pH 5.0, with *p*-NPP plus suitable concentrations of different metal ions, the activity of PchP measured in the presence of Zn^2+^ or Cu^2+^ was higher compared to that with Mg^2+^. Classic kinetic experiments such as saturation curves with substrate or metal ions led to the conclusion that with the *p*-NPP substrate, the catalytic efficiency of PchP activated by Zn^2+^ is approximately 2.5- or 3.2-fold more efficient than with Cu^2+^ or Mg^2+^, respectively. The *K*_M_ value of *p*-NPP was found to be identical when the enzyme was measured with Mg^2+^ or Zn^2+^ but increased approximately 3.5-fold in the presence of Cu^2+^. However, the *K*_A_ value indicated that Zn^2+^ and Cu^2+^ have higher affinity than Mg^2+^ for the metal site of PchP (0.01 mM, 0.02 mM, and 0.6 mM for Zn^2+^, Cu^2+^, and Mg^2+^, resp.) [26]. These results with wtPchP led to experiments designed to obtain kinetic parameters with different mutants. Although different catalytic constants were obtained, no appreciable difference with respect to the activation produced by different divalent cations was detected. Other divalent cations such as Mn^2+^, Co^2+^, and Cr^2+^ (experiments were performed in strict conditions to avoid the oxidation of Cr^2+^ to Cr^3+^) also activated acid phosphatase activity. The results obtained by replacing Zn^2+^ with Co^2+^ will be useful for the study of different physicochemical properties through the utilization of electronic spectroscopy of Co^2+^-PchP derivatives. The catalytic site of PchP lacks both histidine and cysteine, two amino acids that contribute nitrogen or sulfur atoms to coordination complexes in many Zn^2+^- or Cu^2+^-dependent enzymes that generally form tetrahedral or square planar complexes, respectively [44–47]. The utilization of the METALDETECTOR program [48] also indicated that the probability that either histidine or cysteine bound to the metal ion was very low (less than 10% [49]). After this information our focus was placed on the idea that Zn^2+^, Cu^2+^, and Mg^2+^ may form octahedral coordination complexes with the oxygen atom of the carboxylic group of aspartic acid and the main chain carbonyl oxygen. Therefore, we concluded that the three metal ions form an octahedral complex with electron pairs from six oxygen atoms from the two –COO–, one (O*δ*_1_) (^31^D), one (O*δ*_2_) (^262^D), one carbonyl (^33^D), one phosphate (O_1_–P), and two water molecules [25, 26, 38]. All of these donor groups are Lewis bases, and the metal ions are Lewis acids. Pearson [50, 51] introduced the concept of “hardness,” (*η*) “*The nonchemical meaning of the word “hardness” is resistance to deformation or change;*” this concept is useful for explaining the reasons why Cu^2+^ and Zn^2+^ are better activators than Mg^2+^. Chemically speaking, “*hardness is resistance of the chemical potential to change in the number of electrons”* [52]. This author also introduced the concept of chemical hardness and softness in connection with the behavior of Lewis acids and bases, adding, *“hard acids prefer to coordinate to hard bases and soft acids to soft bases.”* Therefore, an acid with hardness *η*_A_ would prefer to bind to a base with similar hardness, *η*_B_. In our case [26], Mg^2+^ is a hard acid (*η*_A_ = 32.5), whereas Zn^2+^ and Cu^2+^ have intermediate values (with *η*_A_ values of 8.3 and 10.8, resp.) [52]. The hardness parameters (*η*_B_) for Lewis bases participating in octahedral complexes are approximately 6 to 7. Because these values are closer to the hardness values of Zn^2+^ and Cu^2+^ and are supported by kinetic data, we conclude that it is easier for these metal ions to form coordination complexes in the active site of PchP than for Mg^2+^. Next, the catalytic mechanism of PchP was studied; Cu^2+^ was discarded for simplicity, and the following experiments were performed with Mg^2+^ and Zn^2+^. The catalytic constant (*k*_cat_) values with *p*-NPP [53] or Pcho [15] as substrates were found to be 1.7- and 1.2-fold higher for Zn^2+^ than for Mg^2+^, respectively. The experiments were performed at pH 5.0 (in acetate buffer) and pH 7.4 (in Hepes buffer) because with Pcho as the substrate and Mg^2+^ as the activator, the optimal pH is a plateau between 5.0 and 8.0 [3]. The first experiments, which were needed to set experimental conditions for the following kinetic studies, were surprising. With Mg^2+^, the same enzymatic activity was measured at pH 5.0 and 7.4. With Zn^2+^, pronounced activity was found at pH 5.0, but no activity was detected at pH 7.4 (Figure 2). We next focused on this lack of PchP activity with Zn^2+^ at neutral or alkaline pH values. The model of the enzyme, including amino acids in the vicinity of motifs I, II, and III, was considered in addition to the ionization of different PchP active site groups involved in the ionization of Pcho and the possible hydrolysis of the metal ion [38]. The model presented in Figure 1(c) shows the hydrophobic amino acid residues located in the *β*-sheets motifs I (^27^YAVF^30^) and III (^257^ILVA^260^). These residues are responsible for creating an environment with a low dielectric constant (*ε*) which according to Dudev and Lim [54] is necessary for the coordination of the metal ion. Kinetic results, obtained at pH 7.4 with saturated Mg^2+^ and low Zn^2+^ concentrations and analyzed with the simulator DYNAFIT [55], indicated that Zn^2+^ might bind to free PchP or PchP-Mg^2+^ complexes with the same affinity to form the nonproductive complex E-Zn^2+^. In addition to this experiment, it has also been shown that inhibition produced by Zn^2+^ in the presence or absence of Mg^2+^ is reversible and dependent on the pH of the reaction. To explain the changes in activity with Zn^2+^ and without Mg^2+^, the ionization state of the enzyme was ruled out as a factor. However, we considered in detail the change of Pcho ionization due to the inactivation of PchP at pH 7.4. We considered the ionization of O-Pcho using the software [ACD, Inc. (http://www.acdlabs.com)] according to the equations

(a) $$\begin{array}{cc} & \text{O=P}\left(\text{O–choline}\right){\left(\text{OH}\right)}_{2}\\ & \;\;⇄\text{O=P}\left(\text{O–choline}\right)\left(\text{OH}\right){\text{O}}^{-}+{\text{H}}^{+},\;\text{p}K_{2}≅6.0 \end{array}$$

(b) $$\begin{array}{cc} & \text{O=P}\left(\text{O–choline}\right)\left(\text{OH}\right){\text{O}}^{-}\;\\ & \;\;⇄\;\text{O=P}\left(\text{O–choline}\right){{\left(\text{O}\right)}_{2}}^{=}+{\text{H}}^{+},\;\text{p}K_{3}\gg 8.0 \end{array}$$

**Figure 3:**
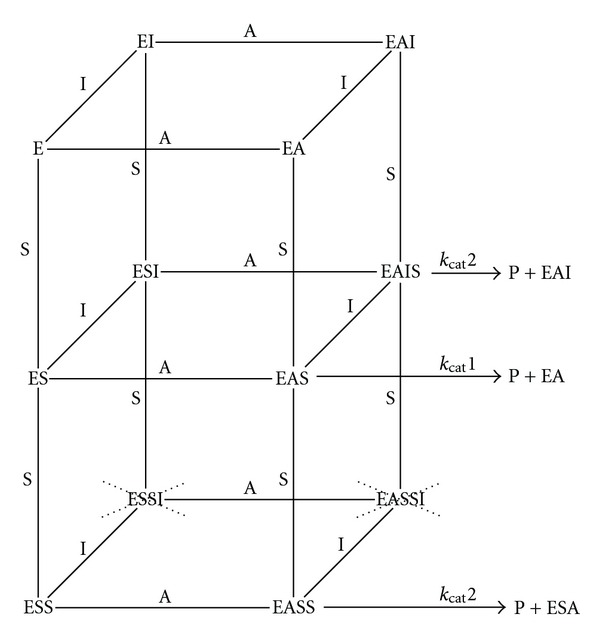
Scheme of the catalytic mechanism of PchP in the presence of metal ion (A), Pcho (S), and AAC (I). All reactions that occur in the cubes are reversible. Different true *K*_M_, *K*_A_, *K*_I_, *K*_SI_, and *k*_cat_ values are published elsewhere [15]. Kinetic data does not indicate that ESSI and EASSI complexes reformed.

Equation (b) was discarded, and (a) was used. The Henderson-Hasselbalch equation (pH = p*K*a + log [A^−^]/[HA]) was used for pH 5.0, 7.4, and pH was set to the p*K*_2_ value. According to (a):

at pH 5.0; 5.0 = 6.0 + log [A^−^]/[HA] and results in the following: [A^−^]/[HA] = 0.1,
at pH 6.0; 6.0 = 6.0 + log [A^−^]/[HA] and results in the following: [A^−^]/[HA] = 1,
at pH 7.4; 7.4 = 6.0 + log [A^−^]/[HA] and results in the following: [A^−^]/[HA] = 25,

at pH 7.4, the main ionic species [A^−^] = [O=P(O–choline)(OH)O^−^] is approximately 250 times more concentrated than at pH 5.0. Under the assay conditions, this anion (free in solution or as a substrate bound to the enzyme) is neutralized by monovalent cations present in the reaction buffer (Na^+^ or K^+^ at pH 5.0 or 7.4). This is true when enzyme's activity is measured in the presence of Mg^2+^ or Zn^2+^. The protonation/deprotonation of O-phosphocholine at different pH values does not change in the presence of Mg^2+^ or Zn^2+^. Therefore, it was assumed that the inhibition produced by Zn^2+^ is caused by an intrinsic property of this ion. The catalysis produced by PchP in the metal ion binding site to form either an octahedral complex [Mg^2+^L_2_^−1^L_2_^0^(H_2_O)_2_] or [Zn^2+^L_2_^−1^L_2_^0^(H_2_O)_2_] is formed by Me^2+^-two COO^−^ (provided by ^31^D and ^262^D residues), -one C=O (provided by ^33^D), -one O=P (from O=P(O–choline)(OH)O^−^ or O=P(O-choline)OH_2_), and the oxygen atoms from two molecules of water. The charge on O=P(O–choline)(OH)O^−^ was ignored because it is not part of the coordination sphere of the metal ion. After these considerations, the results were explained considering the molecular model of the PchP active site [25, 26, 38] in addition to previously described data and theoretical calculations [44, 45, 56–64] related to the coordination of the metal ion with water and their hydrolysis at different pH values, the interaction of carboxylic and carbonyl groups, the substitution of metal ions in the active site, and the hydrophobic cavity with a low *ε* proximal to the active site of PchP. The inhibition produced by Zn^2+^ at pH 7.4 may be interpreted as the change from octahedral to tetrahedral coordination geometry, which is produced by the hydrolysis of the [Zn^2+^L^−1^_2_L^0^_2_(H_2_O)_2_] complex. Zn^2+^, which has an octahedral coordination at pH 5.0 and forms a complex with a charge of zero [Zn^2+^L^−1^_2_L^0^_2_(H_2_O)_2_], may change to a negatively charged complex of either [Zn^2+^L^−1^_2_L^0^_2_(OH)^−1^(H_2_O)]^−1^ or [Zn^2+^L^−1^_2_L^0^_2_ (OH)^−1^_2_]^−2^ at pH 7.4. Therefore, the loss of catalytic activity at pH 7.4 may produce changes in the coordination geometry at the metal binding site of PchP from an octahedral (active enzyme) to a tetrahedral (inactive enzyme) arrangement. Contrary to what occurs with Zn^2+^, the neutral complex of [Mg^2+^L^−1^_2_L^0^_2_(H_2_O)_2_] that forms an octahedral complex does not change between pH 5.0 and 7.4. The consequence is that at both pH values, the PchP activity is similar when Mg^2+^ is utilized as an activator ion [38] (Figure 2). An interesting point emergent of our results is to emphasize that the presence or absence of activity in enzymes of the HAD superfamily may be caused by the hydrolysis of the metal produced by variation of the pH in the reaction mixture. For example, an enzymatic activity measured at neutral or alkaline pH can be active when Mg^2+^ is present but can be inactivated by Zn^2+^ or other ions belonging to the transition metals. As above indicated, the another point of interest was to understand the catalytic mechanism of PchP with Pcho as the substrate, Mg^2+^ or Zn^2+^ as activators, and the effect produced by AACs. Saturation curves with different concentrations of Pcho analyzed in the DYNAFYT software have led to the conclusion that the catalytic mechanism follows a random sequential mechanism for the binding of Pcho, Mg^2+^, or Zn^2+^ (Figure 3) [15]. However, these ions do not change the *K*_M_ value of Pcho. The noteworthy difference between these ions is that the *K*_A_ value for Zn^2+^ is approximately one thousand times lower than the values obtained with Mg^2+^. Another difference is that Zn^2+^ is more effective than Mg^2+^ at avoiding the inhibition produced by high Pcho concentrations. A random mechanism also occurs for the interaction of a second substrate molecule at the second site of the enzyme, which apparently has an affinity for the choline moiety of the substrate or another AAC. These mechanisms are independent of the characteristics of the central metal ion; however, Zn^2+^ is more effective than Mg^2+^ at alleviating the inhibition produced by the entry of the second Pcho molecule or different AACs [15]. This result suggests that Zn^2+^ induces a conformational change in the active center that is communicated to the peripheral anionic site and produces a compact or closed structure. In contrast, a relaxed or open conformation occurs when Mg^2+^ acts as the metal ion activator of the enzyme. To confirm the presence of a second site for the alkylammonium moiety in the PchP molecule, the following compounds were utilized: trimethylamine, tetramethylammonium, choline, chlorocholine, betaine, hexamethonium, decamethonium, tubocurarine, and neostigmine. All these compounds produce inhibition with both competitive and partially uncompetitive components. The presence of two *K*_i_ values also indicates that these compounds, independent of the degree of inhibition produced (the changes produced for the nonalkylammonium moiety present in the inhibitor), might bind to two sites of PchP (e.g., for tetramethylammonium, the *K*_i_1 and *K*_i_2 values are 0.17 and 0.035 mM, respectively) [15]. The concordance between these results and the above-mentioned bioinformatic studies led to the proposal that PchP contains an active site with subsites where the metal ion, the phosphate, and the trimethylammonium moieties are bound. The inhibitory site responsible for the inhibition by high substrate concentrations or different AACs could be a peripheral site located in the vicinity of the active site (Figures 1(b) and 1(c)).

## 5. Biophysical Properties and Crystallization of PchP

**Figure 4:**
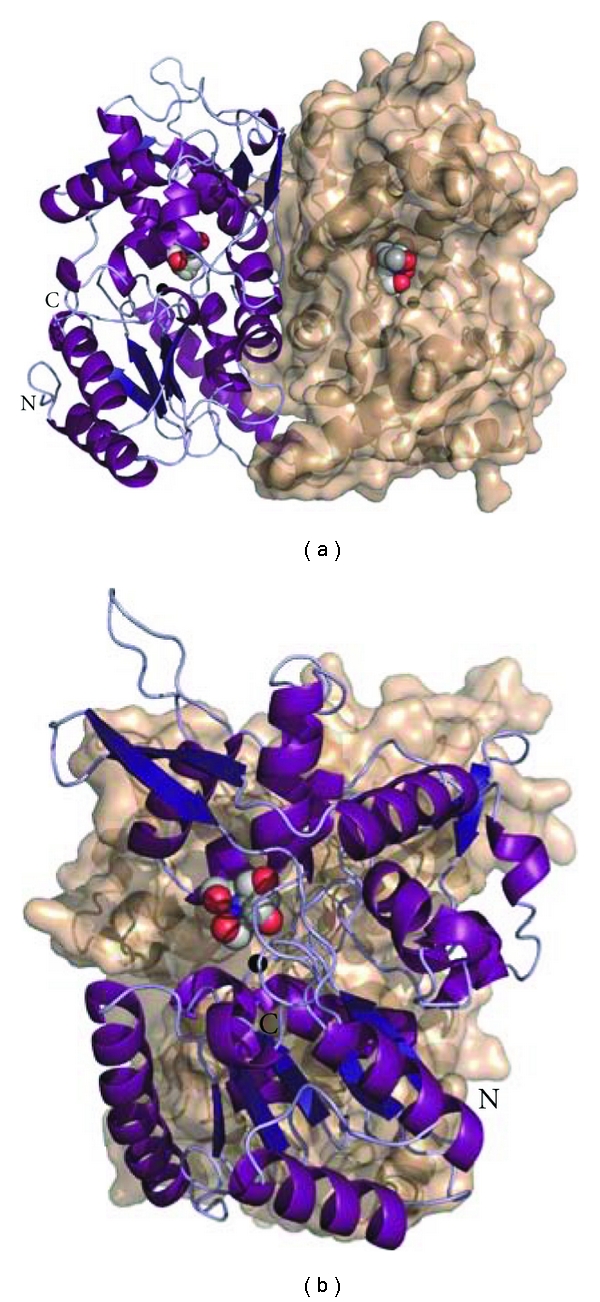
Two perpendicular views of a ribbon representation of the dimeric PchP crystallographic structure. The atoms at the active site are displayed in a spherical mode. The preparation of the figure from crystallographic data was performed by Lourdes Infantes and Armando Albert from IQFR-CSIC, Madrid, Spain.

Simultaneously, attention was focused on studying the physicochemical properties of PchP to facilitate crystallizing the enzyme. The first approach to obtaining enough enzyme to achieve its crystallization and to study its physicochemical properties was performed as previously described [53]. The enzyme was purified from urea, and the inclusion bodies were solubilized and refolded by dialysis. The refolded PchP consisted of a mixture of native PchP and an alternatively folded enzyme aggregate that was slowly converted to the native state. It was proposed that the active enzyme is a dimer, and the catalytic parameters of native PchP for the hydrolysis of *p*-NPP were found to be in excellent agreement with the values reported previously for PchP expressed in *Escherichia coli* as an N-terminal fusion to intein or a histidine tag and purified in the folded state [25, 26, 53]. Although some data were collected, the preparation was not useful for obtaining crystals capable of diffracting. Therefore, efforts were focused on obtaining the enzyme as previously described [38]. Using this preparation technique, crystals were obtained [65], and the crystallization of PchP in the presence of different ligands will be used to solve its structure and to reveal the catalytic mechanism and the possible conformations produced by the different ligands. A structure of the enzyme has recently been obtained and is shown in Figure 4.

## 6. Other Microorganisms Containing PchP

**Figure 5:**
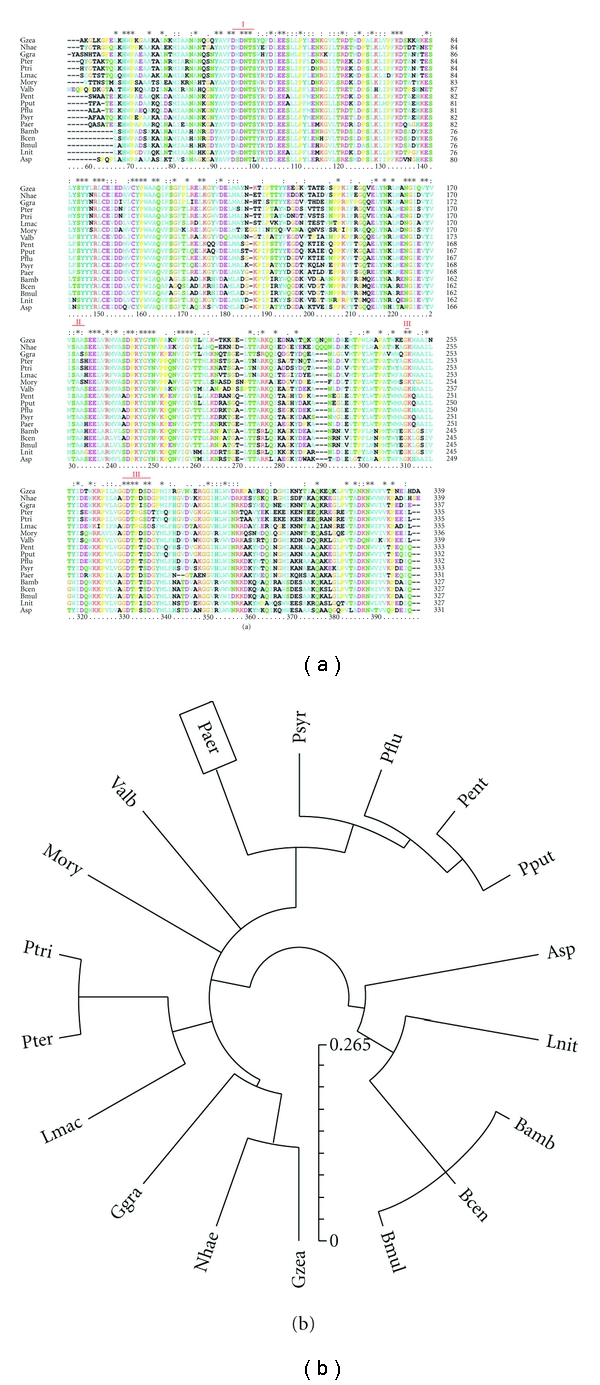
(a) Amino acid sequence alignment of PchP with proteins from different organisms. The sequences analyzed were as follows: Paer, *Pseudomonas aeruginosa* PAO1; Psyr, *Pseudomonas syringae pv. tomato str. *DC3000; Pflu, *Pseudomonas fluorescens* Pf-5; Pent, *Pseudomonas entomophila* L48; Pput, *Pseudomonas putida *KT2440; Asp, *Azospirillum sp.* B510; Lnit, *Lutiella nitroferrum* 2002; Bamb, *Burkholderia ambifaria* MC40-6; Bcen, *Burkholderia cenocepacia* MC0-3; Bmul, *Burkholderia multivorans* ATCC 17616; Gzea, *Gibberella zeae* PH-1; Nhae, *Nectria haematococca* mpVI 77-13-4; Ggra, *Glomerella graminicola* M1.001; Lmac, *Leptosphaeria maculans*; Pter, *Pyrenophora teres* f. teres 0-1; Ptri, *Pyrenophora tritici-repentis* Pt-1C-BFP; Mory, *Magnaporthe oryzae* 70-15; Valb, *Verticillium albo-atrum* VaMs.102. The alignment was constructed using the program CLUSTAL-X [19]; (∗) indicates identical residues, (:) indicates conserved residues, and (.) indicates semiconserved residues. The three catalytic motifs of the HAD superfamily are marked in red. (b) Evolutionary relationships of 18 taxa (linearized). The evolutionary history was inferred using the neighbor-joining method [20]. The optimal tree with the sum of branch length = 2.68745267 is shown. The phylogenetic tree was linearized assuming equal evolutionary rates in all lineages [21]. The clock calibration to convert distance to time was 0.002 (time/node height). The tree is drawn to scale, with branch lengths in the same units as those of the evolutionary distances used to infer the phylogenetic tree. The evolutionary distances were computed using the Poisson correction method [22] and are in units of amino acid substitutions per site. All positions containing gaps and missing data were eliminated from the dataset (complete deletion option). There were a total of 338 positions in the final dataset. Phylogenetic analyses were conducted in MEGA4 [23].

Before the PA5292 gene in the *P. aeruginosa* PAO1 genome was found to be responsible for the synthesis of PchP [4], this protein was known as a hypothetical protein. This enzyme is also present in *P. fluorescens, P. putida, and P. syringae* [49, 66]. The PchPs found in these bacteria were also activated by Mg^2+^, Zn^2+^, and Cu^2+^; Zn^2+^ was the best metal activator of all these enzymes. The kinetic data and the *K*_A_ values for the metal ions defined two groups; one of the groups is formed by *P. aeruginosa* and *P. putida* and has a low affinity for Mg^2+^ with apparent *K*_A_ values of 130 *μ*M and 190 *μ*M, respectively, and the other group is formed by *P. fluorescens* and *P. syringae, *which have a high affinity for Mg^2+^ with apparent *K*_A_ values of 38 *μ*M and 30 *μ*M, respectively. Catalytic efficiency is defined as the relationship *V*_max _/*K*_M_; in this respect, the enzyme isolated from *P. fluorescens* is better at catalyzing the hydrolysis of *p*-NPP in the presence of Mg^2+^, Zn^2+^, or Cu^2+^ with *V*_max _/*K*_M_ values of 2.3, 2.5, and 3.4 for Mg^2+^, Zn^2+^, and Cu^2+^, respectively, followed by those of *P. aeruginosa* and *P. putida* (*≅*0.3–0.4 Umg^−1^mM^−1^ for the three metals) and finally by *P. syringae* (*≅*0.03–0.04 Umg^−1^mM^−1^, for the three metals) [49]. Finally, information from many genomes utilizing the precomputed BLAST results for “http://www.ncbi.nlm.nih.gov/protein/15600485 phosphorylcholine phosphatase [*Pseudomonas aeruginosa* PAO1]” matched 351 proteins in 173 species (331 bacteria, represented principally by *Pseudomonas* and *Burkholderia *species such as *P. aeruginosa*, *P. fluorescens*, *P. syringae*, *P. savastanoi*, *P. entomophila*, *P. putida*, *B. ambifaria*, *B. cenocepacia*, *B. multivorans*, and other bacteria such as *Azospirillum sp*, and *Lutiella nitrofe*). PchP activity has also been found in plant pathogenic fungi such as *Glomerella graminicola*, *Leptosphaeria maculans*, *Pyrenophora teres, P. tritici*, *Gibberella zeae*, *Nectria haematococca*, *Verticillium albo-atrum*, *Magnaporthe oryzae*, and *Phaeosphaeria nodorum *[67]. A multiple alignment of PchP found in different organisms utilizing Clustal X [19] is shown in Figure 5(a); evolutionary relationships and a phylogenetic tree produced by the software [20–23] are shown in Figure 5(b). Because all of these organisms might contain PchP with similar properties to the enzyme found in *P. aeruginosa,* and the crystallized enzyme in the presence of different ligands will soon be solved, the global approach used by our group to study PchP will culminate with the determination of its structure. We expect that further studies might be applied to the development of new and very effective inhibitors of PchP through metal binding, phosphate binding, or alkylammonium binding levels.
